# Beyond Expectations: A Case of Doxorubicin-Induced Cardiomyopathy at a Remarkably Low Cumulative Dose

**DOI:** 10.7759/cureus.42217

**Published:** 2023-07-20

**Authors:** Nisij Shrestha, Prasun Rajbhandari, Sujit Kumar Mandal, Sarosh Janjua

**Affiliations:** 1 Internal Medicine, Upstate University Hospital, Syracuse, USA; 2 Internal Medicine, Dr. Iwamura Memorial Hospital, Bhaktapur, NPL; 3 Internal Medicine, Nepalese Army Institute of Health Sciences, Kathmandu, NPL; 4 Cardiology, Upstate University Hospital, Syracuse, USA

**Keywords:** ejection fraction, diffuse large b lymphoma, cumulative dose, cardiomyopathy, doxorubicin

## Abstract

Anthracycline and its associated cardiotoxicity have been well-established in the literature. With decades of use of anthracycline for a variety of cancer treatments and increased cancer survivability, a detailed study on its cardiac effects is in the continuum. Higher doses of anthracyclines were previously considered the only responsible factor for cardiomyopathy, leading to congestive heart failure. These concepts are now gradually changing to subclinical cardiac changes that even occur at a dosage of 450 mg/m^2^ or less, which was considered safe previously. Here, we present a case of a 64-year-old patient who was started on doxorubicin and then developed subclinical cardiomyopathy at a surprisingly low cumulative dose of 113 mg/m^2^. Hence, this case highlights the importance of exploring risk factors, establishing investigations to pick up early changes, and reconsidering a safe dose of anthracycline on a case-to-case basis.

## Introduction

Doxorubicin, derived from Streptomyces peucetius, has been a cornerstone chemotherapeutic agent for leukemia, lymphoma, and other multiple solid tumors since its discovery in the 1960s. From the time of its regular use in treating numerous cancers, cardiac toxicity has been a matter of primary concern. One of the earliest studies done in 399 patients that brought light to doxorubicin-induced cardiomyopathy showed 11% of patients having ECG changes related to cardiomyopathy and two cases of mortality due to biventricular failure in cancer patients who had no history of preexisting cardiac failure [1].

Doxorubicin has been considered to cause heart failure in up to 38% of people with a cumulative dose of 450 mg/m^2^ and up to 65% at a cumulative dose of 550 mg/m^2^. The cumulative dose of more than 450 mg/m^2^ of doxorubicin has been considered the most crucial cause of cardiotoxicity. Advances in cancer management therapies and an aging population have led to a growing number of cancer patients with comorbid cardiovascular risk factors, which include hypertension, diabetes mellitus, advanced age, smoking, and dyslipidemia. However, gender and the type of cancer are not the risk factor. Doxorubicin administered every three weeks has shown detrimental effects compared to weekly doses [2,3].

Electrocardiographic changes like ST-T segment changes, QRS voltage lowering, T-wave flattening, and QTc interval prolongation, which is non-specific, was previously used as an assessment tool for evaluating cardiomyopathy. However, nowadays, it has been replaced by the measurement of baseline global longitudinal strain on echocardiogram, troponin T, and nuclear stress test [1-3].

## Case presentation

A 64-year white male with more than 45 pack-year smoking history presented with three months history of exertional dyspnea, fatigue, and swelling on the bilateral axilla, neck, and groin. He had significant secondhand smoke exposure since childhood, occasional alcohol use, and no recreational drug use, vaping, or other tobacco use. He did not have any significant past medical history or family history.

On examination, he was found to have non-tender, generalized lymphadenopathy that included the bilateral submandibular lymph node, cervical lymph node, and inguinal lymph node.

His initial lab work is shown in Table 1.

**Table 1:** Initial lab result

Lab Test	Result	Reference Range
White Blood Cell (WBC)	39.8 K	4.5 - 11.0 K/mm³
Neutrophil	50%	40-75%
Lymphocyte	11%	20-45%
Myelocyte	10%	-
Monocyte	8%	2-10%
Lymphoma/Blast	21%	-
Hemoglobin	12.4 g/dL	13.5 - 17.5 g/dL
Platelets	153 K/mm³	150 - 400 K/mm³
Comprehensive Metabolic Panel (CMP)		
Potassium (K)	5.0 mEq/L	3.4 - 5.1 mEq/L
Calcium (Ca)	14.4 mg/dL	8.8 - 10.2 mg/dL
Alanine Transaminase (ALT)	62 U/L	< 41 U/L
Aspartate Transaminase (AST)	269 U/L	< 40 U/L
Alkaline Phosphatase (ALP)	184 U/L	40 - 129 U/L
Blood Urea Nitrogen (BUN)	26 mg/dL	8 - 23 mg/dL
Creatinine (Cr)	1.90 mg/dL	0.7 - 1.2 mg/dL
Lactate Dehydrogenase (LDH)	>2500 U/L	122 - 225 U/L
Uric Acid	13.1 mg/dL	3.4 - 7.0 mg/dL
Parathyroid Hormone (PTH)	6 pg/mL	15 - 65 pg/mL
Peripheral Blood Smear WBC 45.9K (38.5% neutrophil, 24% lymphocytes, 31.5% abnormal lymphocytes, 6% monocytes)		
WBC 45.9K (38.5% neutrophil, 24% lymphocytes, 31.5% abnormal lymphocytes, 6% monocytes)		
Note: The reference ranges provided are general guidelines and may vary depending on the laboratory and specific patient population.		

CT head/neck/chest/abdomen/pelvis without contrast showed multiple bilateral enlarged lymph nodes, most prominent on the right neck measuring 2.0 x 2.5 cm and the largest node in the right submandibular area measuring 2.7 x 1.8 cm; multiple bilateral enlarged axillary lymph nodes, numerous mediastinal and para-aortic/perivascular lymph nodes largest in the pre-carinal area measuring 1.7 cm and most prominent in the axilla on the left side measuring 3.1 cm, 5 mm nodule on the left lower lobe of the lung; large periportal lymph node measuring 3.6 x 5.3 cm, granulomatous lymph node in the retroperitoneal area measuring 13.4 x 4 cm, large right common iliac and external iliac chain lymph node measuring 10.5 x 7 cm, enlarged left common iliac and external iliac chain lymph node, multiple enlarged pelvic lymph nodes and multiple bilateral inguinal lymph nodes.

Left inguinal lymph node excisional biopsy revealed follicular lymphoma, grades 1-2, with a monoclonal, CD10-positive B-cell population. Bone marrow biopsy showed the presence of a high-grade B-cell lymphoma (HGBL) with MYC, BCL2, and BCL6 rearrangements. A baseline echocardiogram (Echo) showed a left ventricular ejection fraction (LVEF) of 65% with normal global systolic function without any wall motion abnormalities, normal left ventricular diastolic function parameters, mildly dilated right ventricle, thickened ventricular wall, and no mention of left ventricular strain.

With curative intent, the patient began treatment on a reduced dose of the R-EPOCH regimen (rituximab, etoposide, prednisone, vincristine, cyclophosphamide, and doxorubicin). The initial cycle consisted of one dose of rituximab at 375 mg/m^2^, five doses of etoposide at 25 mg/m^2^, seven doses of prednisone at 60 mg, five doses of vincristine at 0.2 mg/m^2^, one dose of cyclophosphamide at 562 mg/m^2^, and five doses of doxorubicin at 5 mg/m^2^. The reason for the reduced doxorubicin dose was the patient's slightly elevated liver enzymes. Additionally, the patient received a single dose of intrathecal methotrexate at 12 mg for CNS prophylaxis, along with IV fluids, rasburicase, allopurinol (to address tumor lysis syndrome), a single dose of calcitonin, and a single dose of zoledronic acid to manage hypercalcemia.

After undergoing the first cycle of chemotherapy, he exhibited good tolerance, with positive changes in his blood work. His white blood cell count (WBC) improved to 2.4K, comprising 68% neutrophils, 30% lymphocytes, 1% monocytes, and 1% eosinophils. His hemoglobin level was 8, and his platelet count was 37K. His comprehensive metabolic panel (CMP) showed K at 3.8, blood urea nitrogen (BUN) at 21, Cr at 0.7, Ca at 7.7, alanine transaminase (ALT) at 24, aspartate aminotransferase (AST) at 18, and alkaline phosphatase (ALP) at 63. The uric acid level was measured at 3.1. However, the LDH level remained elevated, exceeding 2500, by the end of the initial chemotherapy cycle.

For the patient's second cycle, the treatment consisted of a single administration of rituximab at a dosage of 375 mg/m^2^, along with four doses of etoposide at 50 mg/m^2^, five doses of prednisone at 105 mg, four doses of vincristine at 0.4 mg/m^2^, a single administration of cyclophosphamide at 750 mg/m^2^, and four doses of doxorubicin at 10 mg/m^2^. These chemotherapy drugs were given every 21 days.

In the third cycle, the patient received a single administration of rituximab at a dosage of 375 mg/m^2^, along with four doses of etoposide at 60 mg/m^2^, five doses of prednisone at 108 mg, four doses of vincristine at 0.4 mg/m^2^, a single administration of cyclophosphamide at 900 mg/m^2^, and four doses of doxorubicin at 12 mg/m^2^. Despite the treatment, the patient continued to experience fatigue and exertional dyspnea, partially attributed to anemia.

The repeat Echo showed LVEF 46% with regional wall motion abnormalities that included the mid anteroseptal wall, mid inferoseptal wall, apical septal wall, mid to apical inferior wall, mid inferolateral wall, and apical lateral walls akinesis, and grade 1 diastolic dysfunction, with no mention of left ventricular strain.

**Figure 1:**
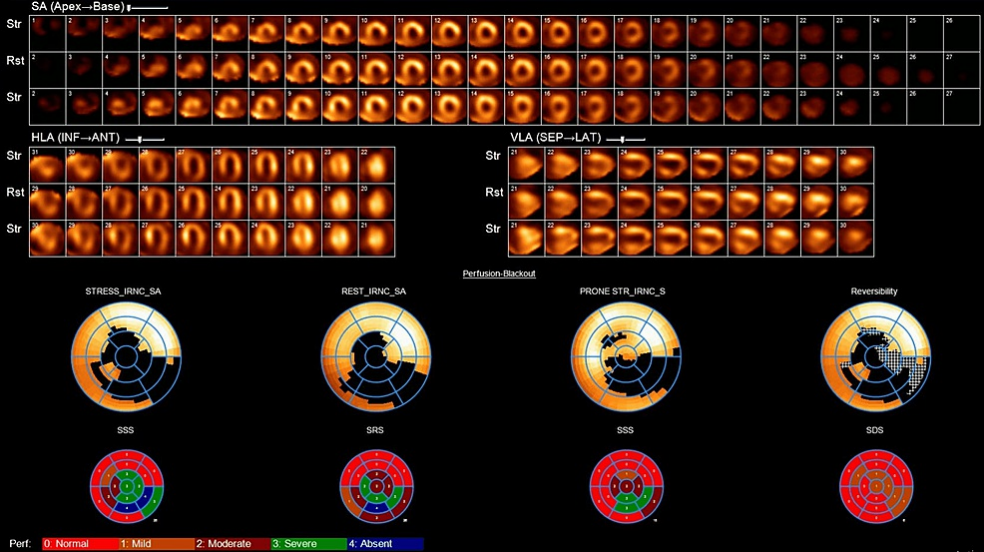
Lexiscan

He denied complaints of chest pain and had no signs of heart failure on examination. High-sensitivity troponin was in the normal range. Given his reduced ejection fraction, he underwent a normal cardiology evaluation, in addition to which an outpatient nuclear stress test was done with Lexiscan (Figure 1), which revealed only an attenuation artifact. Hence, the diagnosis of subclinical cardiomyopathy was made.

Throughout three chemotherapy cycles, he was administered a total cumulative dose of doxorubicin amounting to 113 mg/m^2^. Due to his decreased LVEF, he continued to receive chemotherapy at a reduced dosage.

## Discussion

The safe cumulative dose of doxorubicin has been considered 450 mg/m^2^, below which the congestive cardiac failure risk is low [2]. Given the exponential growth in drug development for cancer therapeutics, it becomes crucial to establish a comprehensive framework for managing cancer therapy-induced cardiomyopathy. This condition is commonly characterized by decreased LVEF of at least 10% to less than 50%. Such a framework is essential to effectively address the cardiovascular risks associated with emerging and established novel cancer therapies. A study done in 141 patients who received average cumulative doses of 300 mg/m^2^ doxorubicin showed subclinical cardiomyopathy in 27.6% of patients, defined as decreased left ventricular fractional shortening (LVFS) by 25% without clinical symptoms or any two of decreased LVFS (28%), decreased LVEF (50%), or abnormal wall motion [4].

Contrary to the dogma, the patient in our case developed asymptomatic cardiomyopathy, a decrease in LVEF by 19% with wall motion abnormality at a comparatively low cumulative dose of 113 mg after three cycles of chemotherapy. The risk of developing cancer therapy-related cardiomyopathy can vary significantly across cancer treatments and is influenced by preexisting cardiovascular risk factors. Patients with cancer and preexisting cardiovascular risk factors are particularly susceptible to cancer therapy-induced cardiomyopathy. Therefore, conducting pretherapy evaluations in this population yields significantly higher benefits. In our specific case scenario, the patient possesses risk factors such as age and smoking history.

Extensive use of doxorubicin in various cancers necessitates providing premeditated dosage and befitting investigations for early detection of adverse effects of the medicine based on the factors influencing its cardiotoxicity.

A meta-analysis revealed an increased risk of clinical cardiotoxicity by 5.43 fold, subclinical cardiotoxicity by 6.25 fold, and cardiac death by 4.94 fold of anthracycline in comparison to the non-anthracycline regimen, with bolus administration being a significant risk factor than continuous low-dose infusion and showed a protective effect of dexrazoxane if used concomitantly [5]. But the patient in our case wasn’t given dexrazoxane, which might have protected him. More prospective trials with dexrazoxane must be done to instill it as a regimen in every individual undergoing anthracycline chemotherapy if found to be significantly protective.

LVEF monitoring has primarily been implemented in patients receiving anthracyclines. In a study involving 2625 patients undergoing anthracycline treatment for breast cancer or lymphoma and who underwent regular LVEF monitoring, approximately 9% of patients developed cancer therapy-related cardiomyopathy. Among those affected, 81% experienced mild symptoms categorized as New York Heart Association (NYHA) classes I to II. Treatment with beta-blockers and angiotensin-converting-enzyme inhibitor-angiotensin receptor blocker (ACEi-ARB) was initiated for all patients, resulting in at least partial recovery of LVEF in 86% of cases. Patients who achieved LVEF recovery demonstrated a lower incidence of cardiac events compared to those who did not [6].

As cardiac function evaluation has been widely limited to measuring LVEF with a risk of interobserver discrepancies, conducting more studies to evaluate other investigations like cardiac troponins and natriuretic peptides for predicting cardiac changes and guiding treatment accordingly is crucial. An increase in troponin T levels as a method of evaluation during serial measurement was highlighted by a study that showed a rise in troponin T in 15% of patients beginning six to 35 days of starting anthracycline therapy that later correlated with a decrease in ejection fraction [7].

A recent study has shown patients who undergo anthracycline therapy have clinically symptomatic heart failure in 2-4%, asymptomatic fall in LVEF in 9-11%, arrhythmia in 12% or more, and cardiac biomarker rise in 30-35% of patients [8]. Therefore, a comprehensive appraisal is required for early diagnosis and preventive and therapeutic management of anthracycline-related cardiotoxicity.

## Conclusions

As in our case, doxorubicin-related toxicity has been seen in an unusually low dose of 113 mg/m^2^, and it has invited a lot of queries regarding risk factors that might not have been on the radar other than age, cumulative dose, and concurrent use of cardiotoxic chemotherapeutic agents. Also, imperative and timely investigations with closer follow-ups would help in the early detection of cardiac insult. An echocardiogram to evaluate for reduced LVEF as a sign of cardiotoxicity alone is a late marker. Further advancements in the field of cardio-oncology necessitate extensive research in various domains. First, an urgent requirement is to establish accurate and universally agreed-upon definitions of cardiovascular toxicity to enhance diagnostic precision. Second, the development of molecular approaches holds immense potential in unraveling the underlying mechanisms of patient susceptibility, allowing for more targeted interventions. In addition, novel cardiovascular strategies must be devised to effectively screen for adverse effects, including the identification of high-risk patient cohorts and the implementation of appropriate monitoring measures. Furthermore, comprehensive clinical trials are indispensable for identifying the most efficacious treatments in cardiovascular toxicity cases and optimizing patient outcomes. Lastly, it is crucial to emphasize the importance of standardized, long-term cardiovascular monitoring in both pediatric and adult cancer survivors, ensuring their ongoing cardiac health and overall well-being.
